# Association Between Severity of Leptospirosis and Subsequent Major Autoimmune Diseases: A Nationwide Observational Cohort Study

**DOI:** 10.3389/fimmu.2021.721752

**Published:** 2021-09-10

**Authors:** Chih-Chung Chen, Yao-Min Hung, Lu-Ting Chiu, Mei-Chia Chou, Renin Chang, James Cheng-Chung Wei

**Affiliations:** ^1^Department of Emergency Medicine, Kaohsiung Veterans General Hospital, Kaohsiung, Taiwan; ^2^Institute of Medicine, Chung Shan Medical University, Taichung, Taiwan; ^3^College of Health and Nursing, Meiho University, Pingtung, Taiwan; ^4^Department of Internal Medicine, Kaohsiung Municipal United Hospital, Kaohsiung, Taiwan; ^5^School of Medicine, National Yang Ming University, Taipei, Taiwan; ^6^Management Office for Health Data, China Medical University Hospital, Taichung, Taiwan; ^7^Department of Physical Therapy, Shu-Zen Junior College of Medicine and Management, Kaohsiung, Taiwan; ^8^Department of Recreation and Sports Management, Tajen University, Pingtung County, Taiwan; ^9^Department of Physical Medicine and Rehabilitation, Kaohsiung Veterans General Hospital, Pingtung Branch, Pingtung County, Taiwan; ^10^Division of Allergy, Immunology and Rheumatology, Chung Shan Medical University Hospital, Taichung, Taiwan; ^11^Graduate Institute of Integrated Medicine, China Medical University, Taichung, Taiwan

**Keywords:** leptospirosis, autoimmune diseases, cohort study, NHIRD, epidemiology

## Abstract

**Introduction:**

Infections play a role in autoimmune diseases (AD). Leptospirosis has been linked to the trigger of systemic lupus erythematosus.

**Objective:**

To investigate subsequent risk of major AD in hospitalized Taiwanese for Leptospirosis.

**Methods:**

Retrospective observational cohort study was employed. The enrolled period was from 2000 to 2012. In the main model, we extracted 4026 inpatients with leptospirosis from the Taiwan National Health Insurance Research Database (NHIRD) and 16,104 participants without leptospirosis at a 1:4 ratio propensity-score matched (PSM) by age, gender, index year, and comorbidities. The follow-up period was defined as the time from the initial diagnosis of leptospirosis to major AD occurrence or 2013. This study was re-analyzed by frequency-matching as a sensitivity analysis for cross-validation. Univariable and multivariable Cox proportional hazards regression models were applied to estimate hazard ratios (HRs) and 95% confidence intervals (CIs).

**Results:**

The adjusted HR (95% CI) of major ADs for the leptospirosis group was 4.45 (3.25–6.79) (p < 0.001) compared to the controls after full adjustment. The risk of major ADs was 5.52-fold (95% CI, 3.82–7.99) higher in leptospirosis patients hospitalized for seven days and above than the controls, while 2.80-fold (95% CI, 1.68–5.61) in those hospitalized less than seven days. The sensitivity analysis yields consistent findings. Stratified analysis revealed that the association between leptospirosis and major ADs was generalized in both genders, and all age groups.

**Conclusions:**

Symptomatic leptospirosis is associated with increased rate of subsequent major ADs, and the risk seems to be higher in severe cases.

## Highlights

**Question**: Is newly diagnosed leptospirosis associated with risk of major autoimmune diseases? Does leptospirosis severity (proxy by length of hospital stay) matter in this issue?
**Findings**: In this Taiwan nationwide cohort study, there was a 4.45-fold increased risk of major autoimmune diseases for newly diagnosed leptospirosis patients, compared with the matched controls. The leptospirosis group had a 6.21-fold higher risk of RA (95% CI 3.09–12.47), 5.15-fold higher risk of SLE (95% CI 1.89–14.53), 7.89-fold higher risk for Sjogren syndrome (95% CI 2.53–24.55), and a 3.72-fold higher risk of Reiter’s syndrome (95% CI 2.22–6.24). The risk was noticeably significant among both genders and all age groups.
**Meaning**: This study demonstrated that leptospirosis was an independent risk factor for developing autoimmune diseases among Asian population. Overall, the risk seems to be higher in the severe cases.

## Introduction

Epidemiological evidence of autoimmune disease (AD) has been found to be steady rising throughout the world over the last decades (1–3). ADs are the third most common category of disease in the United States following cancer and cardiovascular diseases, affecting about 5 to 8% of the population (4). Major ADs are manifested by systemic lupus erythematosus (SLE), rheumatoid arthritis (RA), Sjögren’s syndrome (SS), and Reiter’s syndrome (5). In addition to the environmental and genetic factors that can predispose to the development of ADs, infectious agents also play an important role in the development of ADs (6–9).

Leptospirosis is a human and animal infectious disease that is caused by pathogenic spirochetes of the genus Leptospira (10). It is considered as the most common zoonosis in the world and acknowledged as immunomodulators (11, 12). The role of innate and acquired immunity in host defense to Leptospira remains unclear. Leptospira are able to stimulate various pathways of immune activation. The host immune response crucially influences the outcome of leptospirosis. In humans, T-helper 17 cells have a vital protective role in the immune response to several bacterial and fungal infections (13, 14). Activated Th17 cells can secrete proinflammatory cytokines IL-17 and TNF-α, which drive the regulation of Th17 cell differentiation (15, 16). On the other hand, several studies have disclosed the roles of Th17 cells and IL-17 in major ADs (e.g., SLE, RA, and SS) (17–19). Some studies suggested that elevation of IL-17 and IL-21, IL-23, and TNF-α were involved with Th17 cells in the immune response to Leptospira (20). Leptospira induced significant proliferative responses and cytokine production during acute infection; however, other studies found that there was lack of demonstrable memory T cell in humans recovered from leptospirosis (21). The contribution of Leptospira to reactive arthritis has been published in medical literature (11). Leptospirosis as a possible trigger of SLE was also reported in a medical case (22). However, these reports were either of small case numbers or outbreak series. An earlier study argued that onset of many autoimmune diseases would develop after Leptospira infection (23). They found that patients with prior leptospirosis were at increased risk of subsequently developing many autoimmune diseases (up to 18 autoimmune diseases). However, they included only a small number of measurable relevant covariates in their analysis, so conclusions may be biased, and many of the outcomes of interest accounted for only a small number of events. The study also did not show whether the severity of leptospirosis was involved in the development of major ADs.

Here we conducted a cohort study with more covariates to re-examine the positive link of such association, and we focused on major ADs (e.g., SLE, RA, and SjS) to avoid event competition during follow up (24). Further, in our study, the length of hospital stay for leptospirosis was used to account for the proxy of disease severity.

## Materials and Methods

### Data Source and Study Design

The Taiwan National Health Insurance (NHI) is a universal health insurance system covering more than 99% of the 23 million Taiwan residents. We conducted a population-based matched cohort study using the Longitudinal Health Insurance Database (LHID), a subset of the National Health Insurance Research Database (NHIRD). The NHI records diseases based on the International Classification of Diseases, Ninth Revision, Clinical Modification (ICD-9-CM). Personal identification information in this database is scrambled to protect the privacy of the insured subjects. This study was approved by the Institutional Review Board of China Medical University Hospital Research Ethics Committee [CMUH104-REC2-115(AR-4)].

### Study Population

The study population was comprised of 2 cohorts: the leptospirosis and control cohorts. The leptospirosis cohort consisted of all hospitalized adult individuals with newly diagnosed leptospirosis. These participants were identified by ICD-9-CM code 100.xx from the inpatient claims between 2000 and 2012. To avoid surveillance bias, the control cohort was also extracted from the hospitalization dataset. For leptospirosis patients, the index date was defined as the date of first diagnosis of leptospirosis, and this date was assigned to the matched controls (non-leptospirosis) with the same enrollment criteria. Patients with repeat record of leptospirosis during the follow-up period were not included in the analysis. We established the leptospirosis cohort and the non-leptospirosis cohort by propensity score matching at a 1:4 ratio to observe the incidence of ADs. Major ADs occurred before the index date were excluded: rheumatoid arthritis (RA) (ICD-9-CM code 714), systemic lupus erythematosus (SLE) (ICD-9-CM code 710.0), Sjogren syndrome (SS) (ICD-9-CM code 710.2), and Reiter’s syndrome (ICD-9-CM code 711.0). Participants aged <20 years and those without information on age and gender were also excluded. People were monitored from the index year until occurrence of major ADs listed above, loss to follow-up, withdrawal from insurance, or at the end of December 31, 2013. To reduce heterogeneity and selection bias, both groups have similar distributions of comorbidities with well-balanced PSM.

### Assessment of Baseline Covariates

In this study, we adjusted the statistical model for baseline characteristics and relevant co-morbidities, including diabetes (ICD-9-CM code 250), hyperlipidemia (ICD-9-CM code 272), chronic obstructive pulmonary disease (COPD) (ICD-9-CM code 491, 492, and 496), asthma (ICD-9-CM code 493), cancer (ICD-9-CM code 140–208), allergic rhinitis (ICD-9-CM code 477), atopic dermatitis (ICD-9-CM code 691), and hepatitis B (ICD-9-CM code 070.2, 070.3, and V02.61). These baseline comorbidities were traced back and collected 2 years before the index date.

### Statistical Analysis

The first major AD diagnosis of each patient during the follow-up period was used to calculate the risk of new-onset major ADs. We followed each one of the major ADs to the end of the study to avoid competing censor. The incidence density of major ADs (per 10,000 person-years) was calculated in both groups. We used propensity-scores matching (PSM) to control for sampling bias. Propensity-scores represented patients’ probability of AD incidence, and the scores were determined by a multivariable logistic regression model. The standardized mean difference (SMD) was used to express the difference of the covariates between two groups. If the SMD is less than 0.1, it indicated a negligible difference in the distribution of a covariate between the study and the control groups. To investigate the independent association of leptospirosis with major ADs, Cox proportional hazards model was used to estimate the hazard ratio (HR) and 95% confidence interval (CI). Variables found to be statistically significant in the univariable analysis were further examined in the multivariable model. Adjustment process included age, gender, and comorbidities mentioned above. Kaplan-Meier method was used to plot the cumulative incidence curves and examined the difference of the curves examined by Log rank test.

To validate the robustness of study result, we separately conducted a sensitivity analysis by a different matching method at initial enrollment of participants. The sensitivity analysis was set so that both groups were frequency-matched on age, gender, and index date. Subgroup analyses were conducted in terms of age, gender, comorbidities (grouping as yes or no for all comorbidities), follow-up time, and specific ADs. All statistical tests were 2-sided, and *P* values of.05 or less were considered statistically significant. The statistician used SAS (Version 9.4, SAS Institute Inc., Cary, NC, USA) for all statistical analyses in this study.

## Results

### Clinical Characteristics

In **Table 1**, of 20,130 participants (13,923 males [70%]), 4026 individuals (20%) had newly diagnosed leptospirosis (2823 males [70.12%]); and overall, 127 individuals (0.63%) developed major AD during follow up. In total, 16,104 patients who had no leptospirosis (11,100 males [68.93%]) were matched by age, gender, and comorbidities. The mean ages in the leptospirosis group and non-leptospirosis group were 50.69 ± 18.44 and 50.94 ± 17.80 years, respectively. The follow-up times in the leptospirosis and non-leptospirosis groups were 5.80 ± 3.69 and 5.02 ± 3.08 respectively. The SMD was less than 0.1. As for days spent hospitalized for leptospirosis, **Table 1** showed that in the study group, there was 31.5% patients hospitalized less than 7 days for leptospirosis and 68.5% hospitalized for leptospirosis 7 days and above.

**Table 1:** Baseline characteristics of patients with and without leptospirosis.

	Leptospirosis disease				
	No (n = 16104)		Yes (n = 4026)		SMD
**Characteristics**	**n**	**%**	**n**	**%**	
**Age, years**					
<40	4602	28.58	1149	28.54	0.001
40–60	6376	39.59	1592	39.54	0.001
>60	5126	31.83	1285	31.92	0.001
Mean ± SD	50.69 ± 18.44		50.94 ± 17.80		0.02
**Gender**					
Female	5004	31.07	1203	29.88	0.02
Male	11100	68.93	2823	70.12	0.02
**Comorbidity**					
Diabetes	2321	14.41	582	14.46	0.001
Hyperlipidemia	998	6.20	250	6.21	0.001
COPD	657	4.08	173	4.30	0.01
Asthma	950	5.90	155	3.85	0.09
Cancer	838	5.20	209	5.19	0.001
Allergic rhinitis	125	0.78	26	0.65	0.02
Atopic dermatitis	106	0.66	10	0.25	0.05
Hepatitis B	767	4.76	189	4.69	0.003
**Inpatient days**					
** <7**	NA	NA	1268	31.50	
** >=7**	NA	NA	2758	68.50	
**Follow-up time**	5.80 ± 3.69		5.02 ± 3.08		0.12

Data shown as n (%) or mean ± SD. Chi-square test for categorical data; t-test for continuous data. COPD, chronic kidney disease; SMD, standardized mean difference. A standardized mean difference of 0.1 or more indicates a negligible difference. NA, non-available, not present in this study.

### Occurrence and Relative Risk of Major ADs

In **Table 2**, the overall incidence rates of major ADs in the both groups were 31.62 and 6.30 per 10,000 person-years, respectively. After adjusting for age, gender, and comorbidities, adjusted HR (95% CI) of major ADs for the leptospirosis group was 4.45 (3.25–6.79) (*P* <.001). Patients hospitalized with leptospirosis less than 7 days have aHR of 2.80 (1.68–5.61) (*P* <.001) than the non-leptospirosis group. Patients hospitalized 7 days and above have aHR of 5.52 (3.82–7.99).

**Table 2:** The incidence and HRs for risk of major autoimmune diseases (ADs).

Variables	Autoimmune diseases (n = 127)			Crude HR(95% CI)	Adjusted HR(95% CI)
	Event	PY	IR		
**Leptospirosis**					
No	63	99969	6.30	1 (reference)	1 (reference)
Yes	64	20243	31.62	4.78 (3.37-6.80)***	4.45 (3.25-6.79)***
Inpatient days <7	11	6168	17.83	2.68 (1.41-5.10)***	2.80 (1.68-5.61)***
Inpatient days >=7	53	14075	37.65	5.71 (3.96-8.26)***	5.52 (3.82-7.99)***
**Age, years**					
<40	21	36698	5.72	1 (reference)	1 (reference)
40-60	53	49655	10.67	1.85 (1.11-3.07)*	1.66 (1.01-2.78)*
>60	53	33858	15.65	2.62 (1.58-4.36)***	2.12 (1.24-3.62)**
**Gender**					
Female	55	37560	14.64	1 (reference)	1 (reference)
Male	72	82652	8.71	0.59 (0.41-0.84)**	0.60 (0.43-0.87)***
**Comorbidities**					
Diabetes					
No	96	105844	9.07	1 (reference)	1 (reference)
Yes	31	14367	21.58	2.25 (1.50-3.39)***	1.80 (1.19-2.76)***
Hyperlipidemia					
No	117	113945	10.27	1 (reference)	1 (reference)
Yes	10	6267	15.96	1.48 (0.77-2.83)	1.02 (0.53-1.97)
COPD					
No	114	116316	9.80	1 (reference)	1 (reference)
Yes	13	3895	33.38	3.23 (1.82-5.74)***	2.29 (1.21-4.34)**
Asthma					
No	115	115163	9.99	1 (reference)	1 (reference)
Yes	12	5049	23.77	2.24 (1.24-4.07)**	1.60 (0.82-3.12)
Cancer					
No	124	115782	10.71	1 (reference)	1 (reference)
Yes	3	4429	6.77	0.58 (0.18-1.84)	0.45 (0.14-1.42)
Allergic rhinitis					
No	126	119486	10.55	1 (reference)	1 (reference)
Yes	1	726	13.77	1.22 (0.17-8.73)	0.99 (0.12-6.44)
Atopic dermatitis					
No	125	119807	10.43	1 (reference)	1 (reference)
Yes	2	405	49.38	4.14 (1.02-16.80)*	4.06 (1.08-20.74)*
Hepatitis B					
No	121	115415	10.48	1 (reference)	1 (reference)
Yes	6	4797	12.51	1.13 (0.50-2.58)	1.23 (0.58-3.04)

*p < 0.05, **p < 0.01, ***p < 0.001.

PY, person-years; IR, incidence rate, per 10,000 person-years; HR, hazard ratio; CI, confidence interval.

HR adjusted for age, gender, and each one of comorbidity.

**Figure 1:**
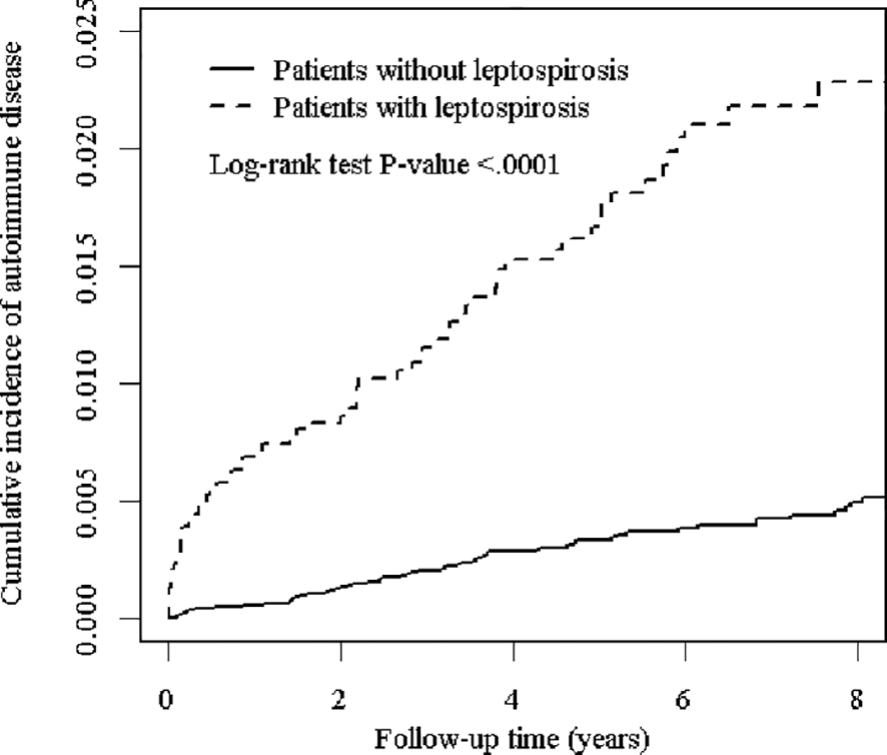
The cumulative incidence of major autoimmune diseases for the patients with and without leptospirosis.

In **Figure 1**, the Kaplan Meier analysis showed that the cumulative incidence of ADs was higher in the leptospirosis group than in the non-leptospirosis group (Log-rank test *P* < 0.001).

### Subgroup Analyses for Age, Sex, and Comorbidities

In **Table 3**, the leptospirosis group was significantly associated with an increased risk of ADs at all age subgroups and both gender subgroups. In age subgroup analysis, leptospirosis group aged <40 years had the highest (aHR, 6.65; 95% CI, 2.76–16.04) risk of developing ADs compared to controls groups with matched age-group of <40 years. Individuals aged 40 to 60 years with leptospirosis had increased risk of developing major ADs (aHR, 4.08; 95% CI, 2.36–7.04). Individuals aged >60 years with leptospirosis also had significantly higher risk of ADs (aHR, 5.28; 95% CI, 3.06–9.13). The *P* value for interaction was non-significant (0.59). In gender subgroup analysis, the leptospirosis group had higher risk of major ADs than the matched controls in both gender subgroups (females-subgroup, aHR, 5.74; 95% CI, 3.34–9.84 and male-subgroup, aHR, 4.50; 95% CI, 2.83–7.18). The *P* value for interaction was non-significant (0.56). Gender difference has no interaction with leptospirosis on the risk of ADs.

**Table 3:** The incidence and HRs for risk of overall ADs in patients with and without leptospirosis stratified by age, gender, and comorbidities.

Variables	Leptospirosis						Crude HR (95% CI)	Adjusted HR (95% CI)
	No (n = 16104)			Yes (n = 4026)				
	Event	PY	IR	Event	PY	IR		
**Age, years**								
<40	9	30353	1.81	12	6345	18.91	6.00 (2.51-14.33)***	6.65 (2.76-16.04)***
40–60	28	41235	3.17	25	8419	29.69	4.13 (2.40-7.13)***	4.08 (2.36-7.04)***
>60	26	28379	11.60	27	5479	49.28	5.26 (3.05-9.08)***	5.28 (3.06-9.13)***
p for interaction								0.59
**Gender**								
Female	26	31403	9.52	29	6156	47.11	5.53 (3.23-9.46)***	5.74 (3.34-9.84)***
Male	37	68565	3.50	35	14086	24.85	4.34 (2.73-6.92)***	4.50 (2.83-7.18)***
p for interaction								0.56
**Comorbidities**								
No	32	73882	4.02	39	14948	22.24	5.29 (3.49-8.95)***	5.79 (3.61-9.28)***
Yes	31	26086	12.45	25	5294	44.44	4.07 (2.38-6.94)***	3.96 (2.32-6.76)***
p for interaction								0.28
**Follow-up years**								
<2	20	30672	6.52	31	7062	43.90	6.66 (3.79-11.67)***	6.70 (3.87-11.95)***
2–5	23	40878	5.63	24	10350	23.19	4.12 (2.32-7.30)***	4.13 (2.33-7.32)***
≥5	20	35197	5.68	9	5245	17.16	2.85 (1.28-6.36)**	3.02 (1.34-6.78)***

**p < 0.01, ***p < 0.001.

PY, person-years; IR, incidence rate, per 10,000 person-years; HR, hazard ratio; CI, confidence interval.

HR adjusted for age, gender and each one of comorbidity. Comorbidity: patients with any one of the comorbidities were classified as the comorbidity group.

We analyzed the association between leptospirosis and the risk of major ADs stratifying by comorbidity and found a 5.79-fold risk of ADs observed in patients without comorbidity (aHR, 5.79; 95% CI, 3.61–9.28) and a 3.96-fold risk of ADs in patients with any comorbidities (aHR, 3.96; 95% CI, 2.32–6.76). The *P* value for interaction was non-significant (0.28).

In term of follow-up years subgroup analysis, patients having leptospirosis had pronounced risk of ADs during the first 2 years of follow up over the matched controls (aHR, 6.70; 95% CI, 3.87–11.95). For participants followed up 2 to 5 years, and more than 5 years, individuals with leptospirosis had significantly higher risk of ADs (aHR, 5.28; 95% CI, 3.06–9.13, and aHR, 3.02; 95% CI, 1.34–6.78 respectively).

### Subgroup Analysis for Specific Major ADs in Taiwan

In **Table 4**, we analyzed the risk of specific major AD. Compared to the non-leptospirosis group, the leptospirosis group had a 7.89-fold higher risk of Sjogren syndrome (95% CI, 2.53–24.55), a 6.21-fold higher risk of RA (95% CI, 3.09–12.47), a 5.15-fold higher risk of SLE (95% CI, 1.89–14.53), and a 3.72-fold higher risk of Reiter’s syndrome (95% CI, 2.22–6.24).

**Table 4:** Incidence and HRs of each AD between patients with and without leptospirosis.

Variables		Leptospirosis disease						Crude HR (95% CI)	Adjusted HR (95% CI)
		No (n = 16104)			Yes (n = 4026)				
		Event	PY	IR	Event	PY	IR		
**Overall ADs**		63	99969	5.32	64	20243	31.62	4.78 (3.37-6.80)***	4.45 (3.25-6.79)***
**Specific Common AD**									
Sjögren’s syndrome	male: 8 (61.5%) female: 5 (38.5%)	5	100201	0.50	8	20408	3.92	7.55 (2.44-23.33)***	7.89 (2.53-24.55)***
Rheumatoid arthritis	male: 13 (39.4%) female: 20 (60.6%)	14	100141	1.40	19	20383	9.32	6.09 (3.03-12.19)***	6.21 (3.09-12.47)***
SLE	male: 14 (93.3%) female: 1 (6.67%)	7	100163	0.70	8	20403	3.92	5.09 (1.84-14.05)**	5.15 (1.89-14.53)**
Reiter’s syndrome	male: 18 (30.0%) female: 42 (70.0%)	34	100097	3.40	26	20345	12.78	3.63 (2.17-6.08)***	3.72 (2.22-6.24)***

**p < 0.01, ***p < 0.001.

PY, person-years; IR, incidence rate, per 10,000 person-years; HR, hazard ratio; CI, confidence interval.

HR adjusted for age, gender, and each one of comorbidity.

### Sensitivity Analysis by Frequency Matching

In the **Supplementary Tables**, the sensitivity analysis yielded consistent findings. The participants were matched by age, sex, and index date and adjusted by hypertension, diabetes, hyperlipidemia, CAD, cerebrovascular disease, CKD, cancer, allergic rhinitis, urticaria, atopic dermatitis, asthma, COPD, sleep apnea, chronic liver diseases, hepatitis B, hepatitis C, splenectomy, alcohol-related illness, and HIV. The aHR for major ADs was 4.86 (95% CI, 3.26–7.26).

## Discussion

The etiology of AD is caused by a complex interaction of environmental-, genetic-, and microorganisms-related factors (25–27). In the cohort study, we discovered an increased risk of major ADs in patients with previous exposure to leptospirosis with an aHR of 4.45 (95% CI, 3.25–6.79; *P* <.001) after adjustment for age, gender, and comorbidities. After cross-validation by two different matching (PS matching and frequency matching), the independent association between leptospirosis and major ADs remained positive, supporting the hypothesis that leptospirosis may trigger or has a role in developing subsequent major ADs. However, the observed link does not indicate causation, and it could be biased by unmeasured confounders.

This study is unique in that the impact of leptospirosis severity on new-onset major ADs has never been approached before. The implications and public health perspectives drawn from this study are of merit. This population-based study allows lots of confounder adjustments and risk stratification by age and gender, which is very important as to the detection and monitoring of the risk groups. The subgroup analysis of our study showed that subjects with leptospirosis in all age groups had increased risks of major ADs. As age increased, the patients with leptospirosis manifested lower risk of developing major ADs in comparison to the control groups. The immune reactions to infections were more intense among younger people than the elders.

Another result deserving our attention is the relatively higher risk of developing major ADs for years after the index date. The risk could persist for years following leptospirosis. One experimental study had shown that using high numbers of Leptospira could induce 10- to 50-fold expansion of gamma/delta T cells and natural killer (NK) cells (28). NK cells have been known to be involved in some autoimmune diseases (29–33), and NK cells can remember prior exposures to become involved for years. A recent study also supported Leptospiral interrogans are associated with innate immune memory (34). The T memory cell of Leptospira infection can persist for up to years, after which the immune dysregulation might recover. Many auto-immunities triggered by infection are more prominent in the first few years.

The underlying mechanism between leptospirosis and risk of ADs remains unclear. Several previous studies showed that acute leptospirosis can trigger the human autoantibodies and lead to pulmonary hemorrhage syndrome (35), uveitis (36), and anti-cardiolipin antibodies (37). Leptospira not only cause acute, systemic infection in animal studies but also persistently colonize and replicate in renal tubules (38), and leptospiral immunoglobulin-like protein A (LigA), relevant to the host immune response to tubulars colonization (39), may involve in the autoimmunity process of the complement deposition and immunoglobulin in the human alveolar basement membrane (40, 41).

One other explanation is that there is increasing evidence of CD8+ T cells playing a role in autoimmunity (42). The activation of CD8+ T cells can be induced and affected by numerous factors, such as inflammatory cytokines, chemokines, and epigenetic modifications (43). Leptospira escaped from human macrophages (44), having peptides complex with MHC class I molecules, could be presented to CD8+ T lymphocytes. CD8+ T lymphocytes specific to LigA protein have been found in human patients (45). Furthermore, Lip L32, one of the Leptospiral outer membrane proteins, could activate Toll-like receptor 2 pathway in human cells to induce innate responses and tubular damage (46, 47). Recent studies have revealed Toll-like receptor 2 to be significant in the pathogenesis of autoimmune diseases (48, 49).

In interpreting our results, several limitations should also be acknowledged. First, due to the retrospective design of the study, causality between leptospirosis and the development of an autoimmune disease cannot be established. Second, NHIRD cannot provide data about personal lifestyle information, family history of autoimmune diseases, or laboratory data. Autoimmune diseases are caused by multiple factors (genetic, environmental) that have not been taken into account because they cannot be controlled, and may therefore act as confounding factors. Patients with asymptomatic or mild symptomatic leptospirosis who never used medical assistance or hospitalization for it were beyond the scope of our study because only patients with symptoms are seen in daily clinical practice. Despite the fact that leptospirosis is a relatively rare disease, it is still possible that participants with mild clinical symptoms of leptospirosis may be included in the control cohort. If leptospirosis is supposed to be positively associated with the subsequent ADs, such misclassification would bias the estimated HRs toward type 2 error (i.e., false negative). Third, the ICD-9-CM codes for the diagnoses of leptospirosis were based on administrative claims data recorded by physicians and hospitals rather than a chart review, which may confound the risk of ADs. However, leptospirosis being prevalent in a tropical area is an important issue as an item list within the document of the Infectious Disease Surveillance and Reporting System in Taiwan. The physicians should conduct a serological test such as microscopic agglutination test-specific antibodies, simultaneously when making a formal “Communicable and Emerging Infectious Disease Report” to Taiwan Centers for Diseases Control. The diagnosis of leptospirosis for inpatients is reliable. However, the laboratory tests results were unavailable in the NHIRD, which is an inherent limitation. Fourth, due to different occurrences of specific ADs among each geographic area, and the study includes mainly Taiwanese population, the results cannot be extrapolated to other populations. There are quite a few autoimmune diseases that have not been included in the study.

## Conclusion

This nationwide longitudinal cohort study demonstrates that leptospirosis is highly correlated with ADs. The risk is consistent in the age-, sex-, and comorbidity-subgroup. Patients with longer leptospirosis-related hospital days are associated with higher risk. We call for more basic research to be conducted in the future to disclose the pathogenic mechanism of ADs through the results of this study.

## Data Availability Statement

The original contributions presented in the study are included in the article/**Supplementary Material**. Further inquiries can be directed to the corresponding authors.

## Ethics Statement

The studies involving human participants were reviewed and approved by Institutional Review Board of China Medical University Hospital Research Ethics Committee (CMUH104-REC2-115(AR-4)). Written informed consent for participation was not required for this study in accordance with the national legislation and the institutional requirements.

## Author Contributions

All authors contributed to the article and approved the submitted version. Study conception and design: C-CC, M-CC, Y-MH, RC, JW. Acquisition of data: L-TC. Analysis and interpretation of data: L-TC, C-CC, M-CC, JW. Writing (original draft preparation): C-CC, M-CC, Y-MH, RC. Writing (review and editing): C-CC, RC, JW.

## Conflict of Interest

The authors declare that the research was conducted in the absence of any commercial or financial relationships that could be construed as a potential conflict of interest.

## Publisher’s Note

All claims expressed in this article are solely those of the authors and do not necessarily represent those of their affiliated organizations, or those of the publisher, the editors and the reviewers. Any product that may be evaluated in this article, or claim that may be made by its manufacturer, is not guaranteed or endorsed by the publisher.
