# Supplementary material for: Association Between Severity of Leptospirosis and Subsequent Major Autoimmune Diseases: A Nationwide Observational Cohort Study
Source: Front Immunol. 2021 Sep 10;12:721752. doi: 10.3389/fimmu.2021.721752 (PMC8461302; doi:10.3389/fimmu.2021.721752)
Supplement: Supplementary file 1 [file Table_1.docx]

**Supplement Table 1.** Baseline characteristics of both groups by frequency matching

|  | **Leptospirosis disease** | | | |  |
| --- | --- | --- | --- | --- | --- |
|  | **No (n=16104)** | | **Yes (n=4026)** | | **p-value** |
| **Characteristics** | **n** | **%** | **n** | **%** |  |
| **Age, years** |  |  |  |  | 0.99 |
| <40 | 4596 | 28.59 | 1149 | 28.54 |  |
| 40-60 | 6368 | 39.62 | 1592 | 39.54 |  |
| >60 | 5109 | 31.79 | 1285 | 31.92 |  |
| Mean ± SD | 50.82±17.87 | | 50.94±17.80 | | 0.71 |
| **Gender** |  |  |  |  | 1.00 |
| Female | 4812 | 29.88 | 1203 | 29.88 |  |
| Male | 11292 | 70.12 | 2823 | 70.12 |  |
| **Comorbidity** |  |  |  |  |  |
| Hypertension | 1564 | 9.71 | 841 | 20.89 | <.0001 |
| Diabetes | 838 | 5.20 | 582 | 14.46 | <.0001 |
| Hyperlipidemia | 423 | 2.63 | 250 | 6.21 | <.0001 |
| CAD | 720 | 4.47 | 311 | 7.72 | <.0001 |
| Cerebrovascular disease | 654 | 4.06 | 318 | 7.90 | <.0001 |
| CKD | 83 | 0.52 | 82 | 2.04 | <.0001 |
| Cancer | 413 | 2.56 | 142 | 3.53 | 0.001 |
| Allergic rhinitis | 33 | 0.20 | 26 | 0.65 | <.0001 |
| Urticaria | 22 | 0.14 | 33 | 0.82 | <.0001 |
| Atopic dermatitis | 11 | 0.07 | 10 | 0.25 | 0.002 |
| Asthma | 227 | 1.41 | 155 | 3.85 | <.0001 |
| COPD | 311 | 1.93 | 173 | 4.30 | <.0001 |
| Sleep apnea | 25 | 0.16 | 11 | 0.27 | 0.11 |
| Chronic liver diseases | 105 | 0.65 | 97 | 2.41 | <.0001 |
| Hepatitis B | 127 | 0.79 | 189 | 4.69 | <.0001 |
| Hepatitis C | 80 | 0.50 | 98 | 2.43 | <.0001 |
| Splenectomy | 11 | 0.07 | 8 | 0.20 | 0.01 |
| Alcohol-related illness | 135 | 0.84 | 250 | 6.21 | <.0001 |
| HIV | 2 | 0.01 | 41 | 1.02 | <.0001 |
| **Follow time** | 5.83±2.80 | | 5.02±3.08 | | <.0001 |

Data shown as n (%) or mean±SD. Chi-square test for categorical data; t-test for continuous data. CAD, coronary artery disease; CKD, chronic kidney disease; COPD, chronic obstructive pulmonary disease; HIV, human immunodeficiency virus.

**Supplement Table 2.** The incidence and HRs for risk of major ADs associated with leptospirosis

| **Variables** | **Autoimmune diseases (n=114)** | | | **Crude HR**  **(95% CI)** | **Adjusted HR**  **(95% CI)** |
| --- | --- | --- | --- | --- | --- |
|  | **Event** | **PY** | **IR** |  |  |
| **Leptospirosis** |  |  |  |  |  |
| No | 50 | 94017 | 5.32 | 1(reference) | 1(reference) |
| Yes | 64 | 20243 | 31.62 | 5.84(4.03-8.45)*** | 4.86 (3.26-7.26)*** |
| **Age, years** |  |  |  |  | 0.99 |
| <40 | 17 | 33930 | 5.01 | 1(reference) | 1(reference) |
| 40-60 | 37 | 46218 | 8.01 | 1.58(0.89-2.82) | 1.35(0.75-2.42) |
| >60 | 60 | 33931 | 17.68 | 3.45(2.01-5.91)*** | 2.75(1.53-4.93)*** |
| **Gender** |  |  |  |  |  |
| Female | 56 | 34519 | 16.22 | 1(reference) | 1(reference) |
| Male | 58 | 79741 | 7.27 | 0.44(0.31-0.64)*** | 0.46(0.31-0.67)** |

*p<0.05, **p<0.01, ***p<0.001

PY, person-years; IR, incidence rate, per 10000 person-years; HR, hazard ratio; CI, confidence interval; HR adjusted for age, gender and each one of comorbidity list in Supplement Table 1.
